# RBF Neural Network-Aided Robust Adaptive GNSS/INS Integrated Navigation Algorithm in Urban Environments

**DOI:** 10.3390/s25237286

**Published:** 2025-11-29

**Authors:** Jin Wang, Ruoyi Li, Rui Tu, Guangxin Zhang, Ju Hong, Fangxin Li

**Affiliations:** 1 College of Geodesy and Geomatics, Shandong University of Science and Technology, Qingdao 266590, China; wangjin@sdust.edu.cn (J.W.); 202383020112@sdust.edu.cn (R.L.); 202283020005@sdust.edu.cn (G.Z.); hongju@sdust.edu.cn (J.H.); lifangxin@sdust.edu.cn (F.L.); 2 Chinese Academy of Surveying and Mapping, Beijing 100830, China

**Keywords:** urban navigation and positioning, GNSS/INS, Robust Adaptive Kalman Filter, RBF neural network, GNSS position increment prediction

## Abstract

Global Navigation Satellite System (GNSS)/Inertial Navigation System (INS) integrated navigation is one of the key methods for achieving precise positioning in complex urban environments. However, in some scenarios such as urban canyons, overpasses, and foliage occlusion, GNSS signals are frequently attenuated or interrupted, leading to degraded positioning accuracy when relying solely on INSs. To address this limitation, this study developed an improved GNSS/INS-integrated navigation algorithm based on a hybrid framework that combines a Robust Adaptive Kalman Filter (RAKF) with a Radial Basis Function (RBF) neural network. The RAKF allows a multi-criterion optimization strategy to be created to adaptively adjust the measurement noise covariance matrix according to GNSS data quality indicators such as PDOP, the number of satellites, and signal quality factors. This enhances the filter’s robustness and outlier detection capability under degraded GNSS conditions. Meanwhile, the RBF network is trained to predict pseudo-position increments, which substitute missing GNSS measurements during signal outages to maintain continuous navigation. Real-world vehicular experiments were conducted to evaluate the proposed RBF-aided RAKF (RBF-RAKF) against three other methods: the Extended Kalman Filter (EKF), standard RAKF, and RBF-aided Kalman Filter (RBF-KF). The experimental results demonstrate that during GNSS outages the proposed method achieved root mean square (RMS) positioning errors of 0.94, 1.02, and 0.21 m in the north, east, and down directions, respectively, representing improvements of over 90% compared with conventional filters. Moreover, the algorithm maintained meter-level horizontal accuracy and sub-meter vertical precision under severe GNSS signal degradation. These results confirm that the proposed RBF-RAKF algorithm provides stable and high-precision navigation performance in challenging urban environments.

## 1. Introduction

As a pivotal technology for vehicle positioning, GNSSs provide continuous or periodic position information, route planning, and time synchronization services to users in open-sky areas [1]. INSs leverage the autonomous positioning capability of Inertial Measurement Units (IMUs) to deliver short-term high-precision navigation during external signal outages. However, they exhibit time-dependent error drift [2,3,4]. Advanced data fusion algorithms such as the Kalman Filter (KF) enable complementary integration of GNSSs and INSs, significantly enhancing navigational accuracy and operational robustness [5].

However, in complex urban scenarios such as urban canyons and overpasses, GNSS signals experience significant degradation [6,7]. The number of satellites decreases substantially, leading to frequent GNSS outages or signal deterioration. This poses two core challenges for integrated navigation systems: (1) the lack of navigation continuity and (2) anomalous measurement noise. When GNSS signal quality deteriorates, the noise covariance assumed by conventional Kalman Filters fails to adapt to dynamically changing observation errors, resulting in filter divergence or position jumping [8]. When GNSS signals are interrupted for an extended period, the accumulated errors in the INS lead to a rapid increase in position drift [9]. Existing solutions typically employ a Robust Adaptive Kalman Filter (RAKF) and interruption compensation techniques. Traditional robust adaptive methods such as the Huber function and interval Kalman filtering rely on empirically set thresholds, making it difficult to dynamically adjust noise covariance, and they suffer from high computational complexity [9]. Commonly used interruption compensation techniques are prediction methods based on kinematic models or sensor fusion, which require additional hardware support, resulting in high costs and insufficient real-time performance [10].

Domestic and international researchers have developed multiple noise estimation strategies and model compensation frameworks, significantly advancing the practical implementation of robust adaptive filtering theory in engineering. For example, Sage and Husa proposed the Sage–Husa adaptive filtering algorithm, which enables online estimation of noise statistical parameters by updating observation data within a real-time sliding window [11]. To address the susceptibility of this method to disturbances from abnormal state equations, scholars have proposed improved solutions which effectively mitigate the impact of abnormal state equations and enhance the algorithm’s stability in complex environments [12,13].

In the Fading Kalman Filter (FKF) proposed by statisticians, a forgetting factor is introduced to dynamically adjust the contribution weight of historical observation data to the current state estimation. This effectively limits the memory length of the Kalman filter, thereby achieving system adaptability [14]. By designing dynamic adjustment factors, a method for dynamically weighting observation data and state prediction information is implemented [15]. To date, various functions for constructing dynamic adjustment factors have been proposed, such as exponential functions [16], logarithmic functions [17], two-segment functions [18], and three-segment functions [19].

Furthermore, to address the Kalman Filter’s (KF) sensitivity to abnormal disturbances during the measurement update process, robust estimation theory has proposed multiple equivalent weight functions with robust characteristics. These include the Huber weight function, Hampel three-segment weight function, Tukey bi-weight function, and IGG III scheme. By establishing a robustified observation value adjustment mechanism, these methods significantly enhance the system’s ability to suppress disturbances caused by outlier data [15,18]. An adaptive method for dynamically adjusting the measurement covariance integrating the Mahalanobis distance criterion and the IGG robust scheme was developed in [20]. This integration improves the robustness of the filtering in degraded environments.

An improved Sage–Husa filter that constructs an innovation-dependent test factor for hypothesis testing was proposed [21], which determines whether real-time estimation of GNSS observation covariance is required while deliberately omitting negative terms in the covariance matrix formulation to ensure positive semi-definiteness. This approach achieves substantial improvement in system stability with minimal loss of navigation accuracy. A Robust Adaptive Kalman Filter based on a non-holonomic kinematic constraint framework that dynamically adjusts robust and adaptive parameters through error sources was also developed [22]. A bidirectional adaptive smoothing filter compensating for system model deviations via forward–backward dual-stage processing with time-varying regulation coefficients was proposed in [23,24]. A hybrid filtering architecture within an interacting multiple model (IMM) framework that significantly enhances robustness and adaptive capabilities was established through the integration of multiple improved Kalman filtering algorithms [25]. An adaptive simplified spherical unscented Kalman filter (ASSUKF) for integrated navigation was introduced in [26]. This approach builds upon the SSUKF framework by integrating an adaptive filter that effectively utilizes residual and innovation sequences to mitigate divergence during the filtering process. Furthermore, the system enables online estimation and dynamic adjustment of measurement noise statistics, achieving more accurate state estimation while significantly enhancing the adaptive capabilities of SSUKF.

Traditional methods struggle to precisely characterize the statistical properties of complex error sources such as multipath effects and signal blockage. Moreover, there is empirical evidence that when GNSS signal degradation coincides with INS error accumulation, singular robust or adaptive strategies fail to simultaneously ensure both robustness and convergence speed in state estimation [27]. Critically, traditional approaches decouple noise covariance tuning through a robust adaptive factor design but they lack a co-optimization framework [28].

Meanwhile, leveraging their strong ability to extract valid information from time-series data, Recurrent Neural Networks (RNNs) have been extensively applied in navigation data processing. Notable implementations include integration with Kalman filters to compensate for GNSS signal outages [29,30]. A hybrid algorithm combining Genetic Algorithm and Support Vector Regression (GA-SVR) was also developed [31]. This method predicts pseudo-position measurements during GNSS unavailability by establishing a nonlinear mapping relationship between IMU outputs (angular velocity and specific force) and satellite-derived position increments. Multi-Layer Perceptron Neural Networks (MLPNNs) and Least Squares Support Vector Machines (LS-SVMs) have been employed to predict pseudo-position measurements for GNSSs [32,33]. The approach incorporated INS velocity navigation solutions as additional input features to the network architecture. In [33], Long Short-Term Memory (LSTM) networks were used to predict GNSS position increments, generating pseudo-position measurements to compensate for missing GNSS signals. The results demonstrated that LSTM outperforms MLPNN solutions during prolonged signal outages. Meanwhile, a methodology integrating Backpropagation Neural Networks (BPNNs) with motion constraint algorithms was developed in [34,35], which further enhances the predicted pseudo-position measurements through odometer-derived constraints. The Gated Recurrent Unit (GRU)—a significant variant of RNNs—was used to construct a pseudo-position measurement generation model for GNSS in [36], thereby aiding adaptive Kalman filtering. A method combining a CNN-Bi-LSTM-Attention model with improved Kalman filtering to enhance robustness and address challenges posed by unknown pseudo-GNSS covariance is presented in [37]. A TransGAN-based INS/GNSS-integrated navigation algorithm was proposed in [38]. This approach constructs generator and discriminator networks, training and optimizing them through adversarial learning to compensate for INS velocity and position errors. This demonstrates TransGAN’s capability to effectively correct velocity and position information with high accuracy during GNSS outages.

The neural network approach for constructing pseudo-observations in GNSS/INS-integrated navigation systems involves predicting GNSS position increments using neural networks and building pseudo-observations through integral operations based on historical data [39]. This processing mechanism inevitably introduces error accumulation effects. Furthermore, neural network prediction models commonly suffer from issues such as high computational complexity, weak online updating capabilities, and susceptibility to prediction outliers [34].

An improved Robust Adaptive Kalman Filtering method for GNSS/INS-integrated navigation, aided by an RBF neural network, is proposed in this paper. This method outputs high-precision integrated navigation positioning results using the improved Robust Adaptive Kalman Filter to enhance positioning performance under poor GNSS signal conditions. The performance gains stem from adaptively optimizing the measurement noise covariance matrix, which is adjusted dynamically based on GNSS data quality indicators such as PDOP, the number of satellites, and the quality factor. Simultaneously, an RBF neural network-based measurement training model is constructed. During GNSS signal outages, pseudo-position increments are predicted using the training model obtained prior to the outage, enabling continuous integrated navigation. This ensures that the system maintains a stable, high-precision positioning capability even in situations of degraded or interrupted GNSS signals.

The remainder of this paper is structured as follows: the GNSS/INS loosely coupled navigation system and the RBF neural network-aided robust adaptive GNSS/INS-integrated navigation algorithm is introduced in Section 2. In Section 3, the experiments for testing the improved Robust Adaptive Kalman Filter are discussed. In Section 4, the road experiments for testing the RBF neural network-aided robust adaptive GNSS/INS-integrated navigation algorithm are discussed. Finally, the conclusions are presented in Section 5.

## 2. Fundamental Principles of the Algorithm

### 2.1. GNSS/INS Loosely Coupled Integrated Navigation System

**Figure 1:**
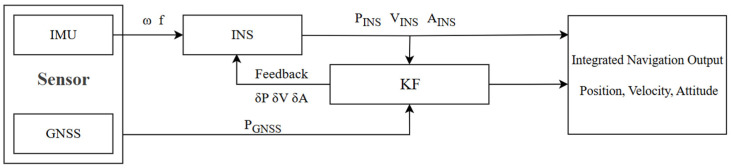
Data fusion process in GNSS/INS loosely coupled navigation systems.

GNSS/INS-integrated navigation systems primarily use three integration modes: loosely coupled, tightly coupled, and deeply coupled. Loosely coupled integrated navigation systems are currently the most widely used integration scheme. The architecture of a GNSS/INS loosely coupled navigation system is illustrated in Figure 1.

For multi-source integrated navigation systems, the discrete-time state model and measurement model can be expressed as [40]

(1) $$\left\{\begin{array}{l} X_{k}=F_{k,k-1}X_{k-1}+G_{k-1}W_{k-1}\\ Z_{k}=H_{k}X_{k}+V_{k} \end{array}\right.$$

where X is the system state vector, Z is the observation vector, F is the state transition matrix, W is the process noise vector, H is the observation matrix. G and V are zero-mean Gaussian white noise processes that are mutually uncorrelated and statistically independent. Subscripts k and k−1 denote discrete time $t_{k}$ and $t_{k-1}$.

(2) $$\left\{\begin{array}{c} {\bar{X}}_{k}=F_{k,k-1}X_{k-1}\\ P_{k/k-1}=F_{k/k-1}P_{k-1}F_{k/k-1}^{T}+G_{k/k-1}Q_{k-1}G_{k/k-1}^{T}\\ K_{k}=P_{k/k-1}H_{k}^{T}{\left(H_{k}P_{k/k-1}H_{k}^{T}+R_{k}\right)}^{-1}\\ \begin{array}{c} {\hat{X}}_{k}=X_{k/k-1}+K_{k}[Z_{k}-H{\bar{X}}_{k}]\\ P_{k}=(I-K_{k}H_{k})P_{k/k-1} \end{array} \end{array}\right.$$

where $P_{k/k-1}$ is the covariance matrix of the state prediction, $K_{k}$ is the Kalman gain of the state update, ${\hat{X}}_{k}$ is the updated state estimate of the state update, $P_{k}$ is the error of the state estimate of the state update, and $Q_{k}$ and $R_{k}$ denote the process noise covariance matrix and measurement noise co-variance matrix, respectively.

The states vector $\delta X\left(t\right)$ is a 21-state vector comprising seven error components: $\delta r^{n}$ (position error), $\delta v^{n}$ (velocity error), a (attitude error), $b_{g}$ (gyroscope bias), $b_{a}$ (accelerometer bias), $s_{g}$ (gyroscope scale factor error), and $s_{a}$ (accelerometer scale factor error). The mathematical formulation is as follows:

(3) $$\delta X\left(t\right)={\left[{\left(\delta r^{n}\right)}^{T}{\left(\delta v^{n}\right)}^{T}a^{T}b_{g}^{T}b_{a}^{T}s_{g}^{T}s_{a}^{T}\right]}^{T}$$

### 2.2. Robust Adaptive Kalman Filter Based on an Improved Measurement Noise Covariance Matrix

#### 2.2.1. Multi-Criterion Optimized Measurement Noise Covariance Matrix

In previous research, GNSS positioning error estimation was typically represented using empirical values or variances [41]. However, in complex urban environments, a significant inconsistency exists between the estimated GNSS errors and actual positioning errors. To address this, this study improved the measurement noise covariance matrix $R_{k}$ through collective weighting of the Position Dilution of Precision (PDOP), the number of satellites, quality factor Q, and position root mean square error r. This approach appropriately inflates $R_{k}$ to reduce the influence of observations on the filter. The criteria for the multi-factor decision were predefined: the threshold for PDOP was set to 2, the standard for the number of satellites was set to 12, and the standard for the quality factor was set to 0 or 1.

The expression for $R_{k}$ is as follows:

(4) $$R_{k}=\mathrm{diag}\left[r^{2}\left(1+\beta \right)\right]$$

**Figure 2:**
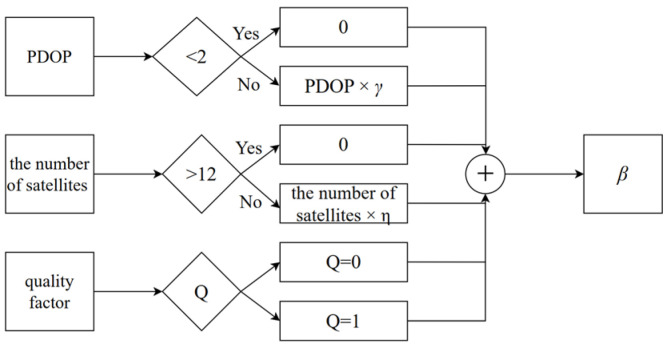
GNSS data quality indicator.

The determination of β is illustrated in Figure 2.

Here, the role of β is to dynamically adjust $R_{k}$ based on PDOP, the number of satellites, and quality factor Q. γ and η are used to quantify the quality of the observation data, which are in the ranges of 0.1~0.5 and 0.01~0.05, respectively. $R_{k}$ can be adjusted within an appropriate range.

#### 2.2.2. Robust Adaptive Kalman Filter Based on an Improved Measurement Noise Covariance Matrix

The Robust Adaptive Kalman Filter (RAKF) was introduced to address the issues when GNSS signals are affected by environmental factors such as obstruction, interference, and reflection, which cause measurement data to deviate from Gaussian distribution assumptions and could potentially cause outliers or anomalies. By adaptively adjusting the noise covariance matrix during filtering, this approach can exhibit strong robustness against outliers and abnormal data.

The robust filtering primarily determines the presence of gross errors in GNSS observations through outlier detection based on innovation discrepancy; it reduces the covariance of observations with higher precision while increasing the covariance of observations with lower precision. Its expression is as follows:

(5) $${\bar{R}}_{k}=\alpha R_{k}$$

where ${\bar{R}}_{k}$ denotes the equivalent covariance matrix of the measurement noise matrix $R_{k}$, and α represents the robustness tuning factor.

This study employed a three-stage robust filtering method based on innovation. According to the joint Gaussian distribution characteristics of state parameters and observed variables, it can be derived that the filter innovation sequence follows the statistical distribution below:

(6) $$v_{k}\sim N(\theta ,H_{k}P_{k,k-1}H_{k}^{T}+R_{k})$$

where $N\left(\mu ,\sum \right)$ is defined as following a normal distribution with mean μ and covariance matrix ∑. Based on this property, standardized residuals that follow a standard normal distribution can be constructed:

(7) $${\bar{\tilde{v}}}_{i}=\frac{v_{i}}{P_{v_{i}}}$$

where ${\bar{\tilde{v}}}_{i}$ denotes the standardized innovation element and $P_{v_{i}}$ represents the standard deviation corresponding to element in the innovation vector, which can be obtained from the diagonal elements of the innovation covariance matrix $P_{v}$.

This study also employed the IGGIII weight function [42]; its mathematical formulation is shown as Equation (8):

(8) $$\alpha_{i}=\left\{\begin{array}{cc} \infty & k_{1}<\left|{\bar{\tilde{\nu }}}_{i}\right|\\ \frac{k_{0}}{\left|{\bar{\tilde{\nu }}}_{i}\right|}(\frac{k_{1}-\left|{\bar{\tilde{\nu }}}_{i}\right|}{k_{1}-k_{0}}) & k_{0}<\left|{\bar{\tilde{\nu }}}_{i}\right|\leq k_{1}\\ 1 & \left|{\bar{\tilde{\nu }}}_{i}\right|\leq k_{0} \end{array}\right.$$

where $\alpha_{i}$ denotes the robustness factor, with the discrimination threshold $k_{0}$ typically ranging from 1.0 to 1.5 and threshold $k_{1}$ ranging from 2.5 to 3.0. Based on this piecewise weight function model, a robustly optimized Kalman gain matrix can be constructed:

(9) $${\bar{K}}_{k}=P_{k/k-1}H_{k}^{T}{\left(H_{k}P_{k/k-1}H_{k}^{T}+\alpha R_{k}\right)}^{-1}$$

Leveraging the principle of robust estimation can mitigate the impact of observation gross errors on filtering results. Beyond this, deterioration of state predictions may also occur due to factors such as the maneuvering complexity of the carrier vehicle, inaccuracies in the dynamic model, and errors from low-cost inertial sensors. To address this, a state prediction residual model must be constructed:

(10) $$v_{x}=X_{k,k}-X_{k,k-1}=X_{k,k}-F_{k-1}X_{k-1,k-1}$$

where $v_{x}$ denotes the state prediction vector residual caused by errors accumulated during the time update phase. Based on this residual, an adaptive factor λ can be constructed, as shown in Equations (11) and (12):

(11) $$\lambda =\left\{\begin{array}{c} \begin{array}{cc} 1 & \left|{\tilde{v}}_{x}\right|\leq c \end{array}\\ \begin{array}{cc} \frac{\left|{\tilde{v}}_{x}\right|}{c} & c<\left|{\tilde{v}}_{x}\right| \end{array} \end{array}\right.$$

(12) $${\tilde{v}}_{x}={\left(\frac{v_{k}^{T}v_{k}}{tr\left(P_{k,k-1}\right)}\right)}^{\frac{1}{2}}$$

The Robust Adaptive Kalman Filter (RAKF) state estimate is as follows:

(13) $$\begin{array}{c} {\bar{\bar{\tilde{K}}}}_{k}=\lambda P_{k/k-1}H_{k}^{T}{\left(H_{k}\lambda P_{k/k-1}H_{k}^{T}+\alpha R_{k}\right)}^{-1}\\ X_{k}=X_{k/k-1}+{\bar{\bar{\tilde{K}}}}_{k}v_{k}\\ {\tilde{P}}_{k}=(I-{\bar{\bar{\tilde{K}}}}_{k}H_{k})P_{k/k-1} \end{array}$$

In conclusion, the multi-factor dynamic adjustment of $R_{k}$ in Equation (4) is implemented to address the time-varying characteristics of GNSS noise, reducing positioning errors caused by fixed estimation bias. The robustness tuning factor α and adaptive factor λ were introduced in Equations (8) and (11). The Robust Adaptive Kalman Filter (RAKF) enhances the robustness of filtering and reduces the impact of gross errors on positioning, particularly under poor GNSS conditions.

### 2.3. RBF Neural Network-Aided Robust Adaptive GNSS/INS-Integrated Navigation Algorithm

In complex urban terrains, obstacles such as high-rise buildings, underground tunnels, and overpasses significantly degrade GNSS signal observability. Under extreme circumstances involving prolonged GNSS signal outages, the positioning errors of GNSS/INS-integrated navigation systems exhibit divergence trends, thus compromising the navigation outputs. Here, the use of a pseudo-GNSS value predicted by an RBF neural network during GNSS outages was proposed.

#### 2.3.1. RBF Neural Network

As a typical feedforward network architecture, RBF neural networks guarantee global optimality and exhibit fast convergence. They are capable of approximating any continuous nonlinear system, making them suitable for applications such as motion trajectory prediction and those involving integrated navigation systems.

The classic RBF neural network forms a complete computational chain comprising three layers: an input layer, a hidden layer, and an output layer. The input feature vector x is passed from the input layer to the hidden layer. Each node in the hidden layer represents a Radial Basis Function (RBF). The RBF is typically a Gaussian function.

The expression of the RBF is as follows:

(14) $$\mathrm{RBF}(x)=e^{-x^{2}}$$

The expression of the hidden layer is as follows:

(15) $$H_{0}=\mathrm{RBF}(b^{\left(2\right)}\times dist(W^{\left(2\right)},x))=\exp(-{\left(b^{\left(2\right)}\right)}^{2}\times {\left|\right|W^{\left(2\right)},x\left|\right|}_{2}^{2})$$

where dist denotes the distance function, typically the Euclidean norm. $W^{\left(2\right)}$ and $b^{\left(2\right)}$ represent the weight and bias term (threshold) of the second layer (hidden layer), respectively.

The output layer calculates a weighted sum of the outputs from all hidden layer nodes using weights $W^{\left(3\right)}$, and adds a bias term $b^{\left(3\right)}$(threshold). This yields the final output of the RBF neural network. The terms $W^{\left(3\right)}$ and $b^{\left(3\right)}$ represent the weight and bias term (threshold) of the third layer (output layer), respectively [43]. The expression of the output layer is as follows:

(16) $$y=W^{\left(3\right)}\times H_{0}+b^{\left(3\right)}$$

**Figure 3:**
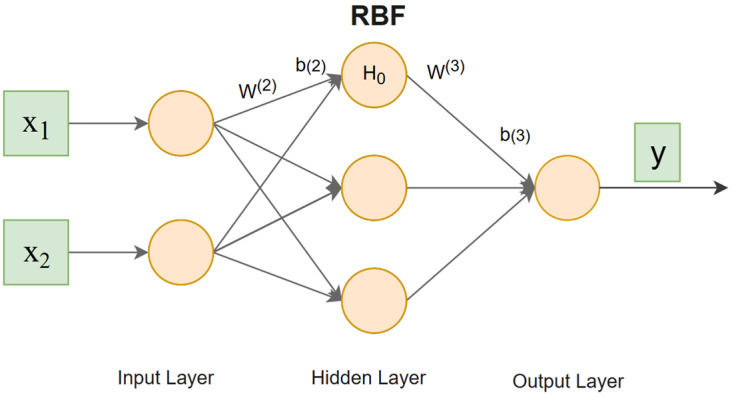
RBF neural network topology diagram.

The structure of the RBF neural network is shown in Figure 3.

The training of an RBF neural network involves determining the weights and centers such that the model’s final output matches the true values. Three common fundamental training methods for radial basis function neural networks are the interpolation method, the Orthogonal Least Squares (OLS) method, and the K-means clustering method. This study employed the K-means clustering method.

#### 2.3.2. RBF Neural Network-Aided Robust Adaptive GNSS/INS-Integrated Navigation Algorithm

**Figure 4:**
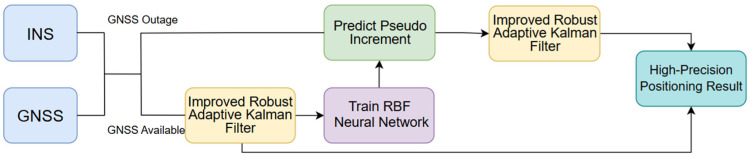
Architecture of RBF-aided integrated navigation.

To address the issue of rapidly diverging positioning errors in GNSS/INS-integrated navigation systems caused by GNSS signal attenuation or even outages in complex urban environments, this study proposes a novel integrated navigation scheme combining an enhanced Robust Adaptive Kalman Filter with an RBF neural network. First, this method utilizes high-precision positioning results output by the Robust Adaptive Kalman Filter algorithm for the GNSS/INS-integrated navigation system. Simultaneously, it constructs an RBF neural network training and prediction model to forecast the pseudo-position increments of GNSS signals during outages. This dual approach ensures that the system maintains stable, high-precision positioning performance even under conditions of temporary GNSS signal loss or severe interference. The overall architecture of the proposed algorithm is illustrated in Figure 4.

The process of the fusion algorithm functions in two distinct modes, as detailed below.

**Figure 5:**
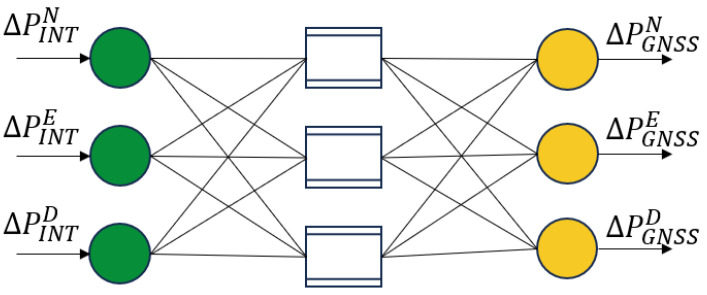
Inputs and outputs of RBF neural network.

(a) When GNSS signals are available:
  ① The Robust Adaptive Kalman Filter is enhanced through improvements in the measurement noise covariance matrix to achieve high-accuracy navigation and positioning results, while simultaneously conducting RBF neural network training.
  ② Input dimension compression is achieved and training efficiency is enhanced by employing $O_{INT}-O_{GNSS}$ as the input–output mapping framework in the network topology design. The position increments $\Delta P_{INT}$ from the integrated navigation system serve as the neural network input, while the GNSS position increments $\Delta P_{GNSS}$ are designated as the target output for training the prediction model. The input and output layers are expressed as follows:
    (17) $$x_{j}=\left[\Delta P_{INT}^{n-j},\begin{array}{c} \ldots \end{array}\Delta P_{INT}^{n-1}\begin{array}{c} , \end{array}\Delta P_{INT}^{n}\right]$$
    (18) $$y_{j}=\left[\Delta P_{GNSS}^{n-j},\ldots \Delta P_{GNSS}^{n-1},\Delta P_{GNSS}^{n}\right]$$
    where $x_{j}$ denotes the position increment output from the GNSS/INS-integrated system during the training period; $y_{j}$ represents the reference position increment from the GNSS navigation system, which is a three-dimensional vector in the north, east, and down components; and j denotes the corresponding sampling epoch. The inputs and outputs of the RBF neural network are shown in Figure 5.
  ③ The centers of the RBF basis functions are determined using the K-means clustering algorithm. Through multiple experimental validations and comprehensive evaluation based on prediction mean squared errors (MSEs) and training time consumption, the number of hidden layer nodes is optimized to three. The neural network preset training target is set to 0.01 with 300 maximum iterations. The training process continuously adjusts weights and expansion factors between the input and output layers, aiming to minimize prediction errors and establish the optimal mapping relationship.
(b) When GNSS signals are unavailable:
  ① Assuming the GNSS outage occurs at epoch t, the position increments $\Delta P_{INS}^{t+1}={\left(\Delta \lambda_{INS}^{t+1},\Delta l_{INS}^{t+1},\Delta h_{INS}^{t+1}\right)}^{T}$ from INS mechanization are fed into the pre-trained RBF neural network model. This generates predicted pseudo-GNSS position increments $\Delta P_{pre-GNSS}^{t+1}={\left(\Delta \lambda_{pre-GNSS}^{t+1},\Delta l_{pre-GNSS}^{t+1},\Delta h_{pre-GNSS}^{t+1}\right)}^{T}$ as the output.
  ② The pseudo-measurements $\Delta P_{pre}$ predicted by the RBF neural network are incorporated into the improved Robust Adaptive Kalman Filter. The final GNSS/INS-integrated navigation positioning result is thus obtained.

The above algorithm integrates Robust Adaptive Kalman Filtering and an RBF neural network to improve the positioning accuracy when the GNSS signal is poor. The pseudo-position increments generated by the RBF neural network replace the missing GNSS values for navigation and positioning during GNSS interruption.

## 3. Robust Adaptive Filter with Improved Measurement Noise Matrix Testing

To validate the effectiveness of the Robust Adaptive Kalman Filter (RAKF) when GNSS signals are poor, a dataset was collected to validate the algorithm. The experimental details are given in this section.

### 3.1. Data Collection

**Figure 6:**
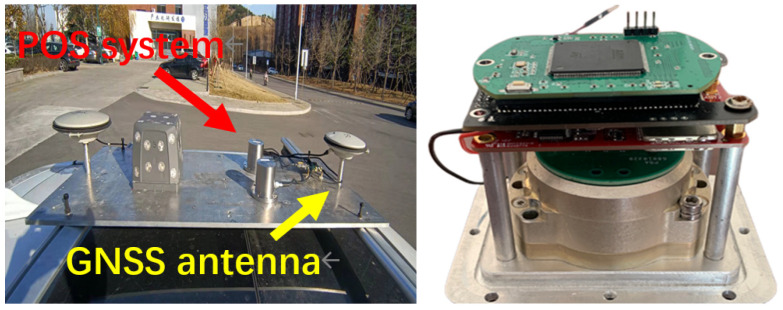
Sensors installed on vehicle.

To validate the effectiveness of the improved robust adaptive filtering, a self-developed POS inertial guidance system was installed on a vehicle to collect inertial navigation data. The self-developed POS system operated at a sampling frequency of 100 Hz. For GNSS data acquisition, an OEM718D GNSS receiver, manufactured by NovAtel in Calgary, Canada with a sampling frequency of 5 Hz was employed. The specifications of the sensors are detailed in Table 1. A GNSS reference station was established on the rooftop of the College of Geomatics and Spatial Information at Shandong University of Science and Technology. The experimental equipment was rigidly mounted on the vehicle to form a rover station. The sensors installed on the vehicle are shown in Figure 6. A single-antenna model was adopted in this experiment (a dual-antenna receiver was mounted on the vehicle platform, but only single-antenna data were used).

**Figure 7:**
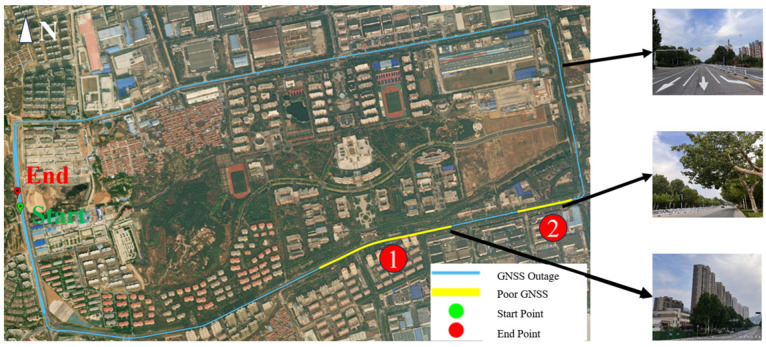
Route and scenes from experiment. Segment ① with high-rise obstruction and Segment ② with tree obstruction.

The experimental data acquisition spanned from 06:22:00 to 06:38:28 on 29 April 2022, lasting approximately 20 min. The test trajectory formed a rectangular route around Shandong University of Science and Technology, encompassing environments with tree canopies, high-rise building obstructions, and open-sky conditions. The route and scenes from the experiment are shown in Figure 7, where the yellow Segments ① and ② indicate trajectories corresponding to abnormal disturbances.

**Figure 8:**
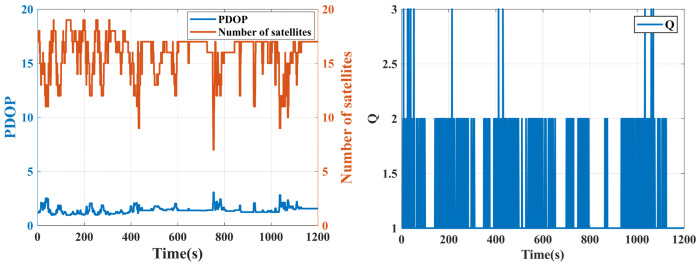
PDOP, number of satellites (**left**), and positioning solution quality (**right**).

The number of satellites and PDOP during this experiment are shown in Figure 8. The number of satellites predominantly fluctuated between 12 and 18, peaking at 19. However, significant drops occurred intermittently—for instance, decreasing to as low as 10 satellites—exhibiting pronounced high-frequency instability. The changes in the number of satellites may be attributed to receiver performance limitations and signal obstructions caused by high-rise buildings and tree trunks along the vehicle trajectory.

The PDOP exhibited minor fluctuations but predominantly maintained favorable levels between 1 and 2. This indicates excellent satellite geometry. However, occasional spikes were observed exceeding this range.

There was an inverse correlation between the number of satellites and PDOP: a higher number of satellites generally yielded a lower PDOP due to improved geometric distribution, enhancing the positioning precision. Conversely, a reduced number of satellites typically elevated PDOP as degraded geometry or insufficient satellites compromised the positional accuracy. As shown in Figure 8, PDOP exhibited minimal fluctuations in most intervals, demonstrating that favorable geometric integrity persists despite variations in the number of satellites. During significant drops in the number of satellites, such as at 750 s when the number of satellites fell below 8, the PDOP manifested a sharp increase, indicating geometric degradation due to signal obstruction.

**Figure 9:**
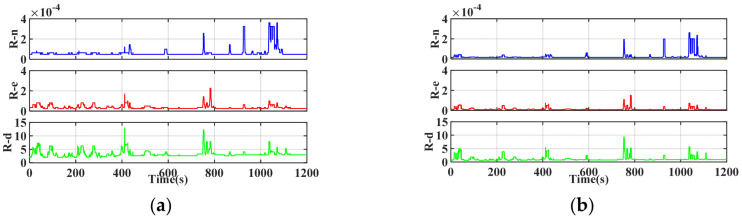

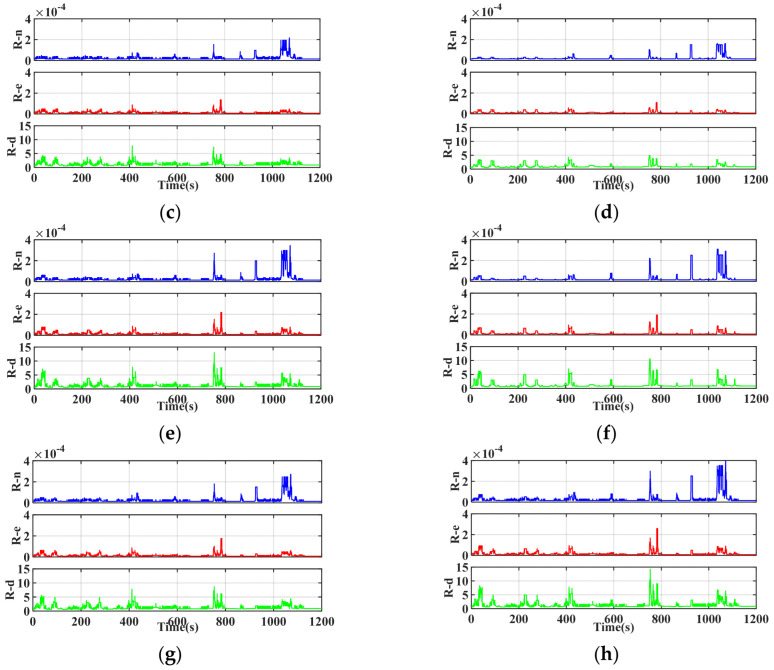
Variation in diagonal elements for measurement noise covariance matrix in the north, east, and down directions.

The composition of eight different observation variances is listed in Table 2, based on the GNSS data duality indicator shown in Figure 2. The raw observed data were processed using the RAKF algorithm through eight different schemes. The temporal evolution of diagonal elements in the experimentally derived measurement noise covariance matrix is shown in Figure 9. Curves 1–8 correspond to the results from distinct parameter schemes, which revealed pronounced variations in the values at approximately 400, 800, and 1000 s.

The high-accuracy navigation solution obtained through kinematic differentials and Inertial Explorer 9.00 software, manufactured by NovAtel in Calgary, Canada served as the reference truth value. The error statistics were computed between the navigation results of the eight schemes and the reference truth value. The RMS of position, velocity, and attitude with different measurement noise covariance matrices is presented in Table 3. Scheme 1 is the traditional EKF algorithmic and the comparison between the EKF and Scheme 8 showed that Scheme 8 achieved improvements in positioning accuracy of 29.32%, 27.27%, and 44.07% in the north, east, and down directions, respectively. Compared to Scheme 2, the accuracy of Scheme 8 improved by 25.76% in the down velocity while maintaining similar positioning, velocity, and attitude accuracies. Relative to Scheme 3, the vertical positioning accuracy of Scheme 8 improved by 4.91% while compared to Scheme 5, Scheme 8 showed significant improvements of 21.89% in down positioning accuracy and 30.70% in down velocity accuracy. Compared to Scheme 6, the positioning accuracy of Scheme 8 increased by 17.13% in the down direction. Finally, compared to Scheme 7, Scheme 8 achieved a 20.54% improvement in down positioning accuracy.

Therefore, considering positioning, velocity, and attitude errors, the optimal selection is Scheme 8.

### 3.2. Experimental Results

To facilitate comparative analysis of different filtering algorithms’ performance in a GNSS/INS-integrated navigation system, four distinct filtering approaches were designed for the experiment. All schemes were based on the Extended Kalman Filter (EKF), incorporating different enhancement strategies to improve the filter’s robustness and adaptability. By comparatively analyzing the filtering results of these four schemes across various scenarios, we can comprehensively evaluate performance differences between the algorithms. This analysis offers theoretical support and experimental evidence for optimizing the design of GNSS/INS-integrated navigation systems.

The four schemes are as follows:

Scheme 1: Extended Kalman Filter (EKF);

Scheme 2: Adaptive Kalman Filter (AKF);

Scheme 3: Robust Kalman Filter (RKF);

Scheme 4: Robust Adaptive Kalman Filter (RAKF) with an improved measurement noise covariance matrix.

**Figure 10:**
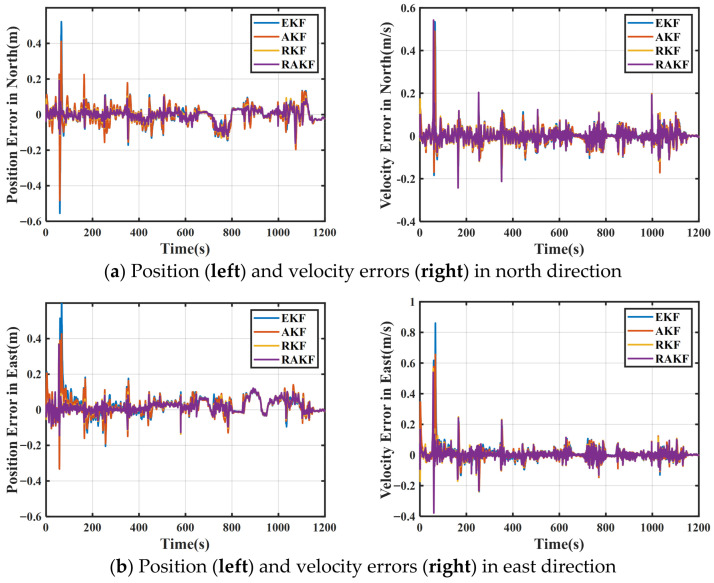

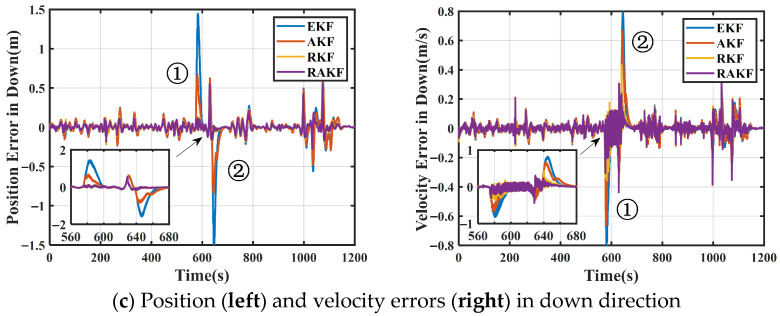
Comparison of position and velocity errors of four different schemes in the (**a**) north, (**b**) east, and (**c**) down directions.

The high-accuracy navigation solution obtained through kinematic differentials and Inertial Explorer 9.00 software served as the reference truth value. The position and velocity errors of the four different filtering schemes in the north, east, and down directions are shown in Figure 10.

The position and velocity errors of the four different schemes in the north, east, and down directions are shown in Figure 10. It can be observed that significant variations in both position and velocity errors occurred at approximately 600 s. A zoomed-in view of this interval reveals that the RAKF scheme exhibited minimal fluctuations in velocity and position errors, with values remaining closer to zero compared to the other three schemes.

**Figure 11:**
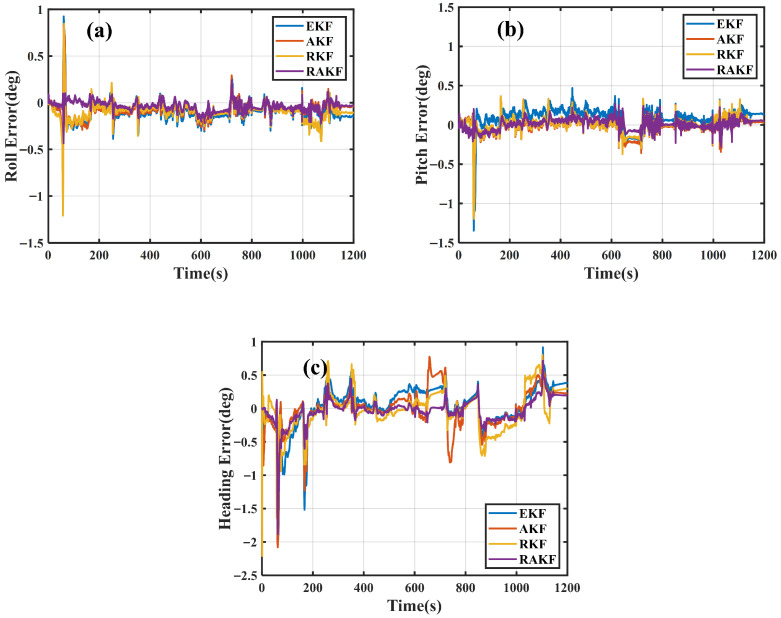
Comparison of attitude errors in (**a**) roll, (**b**) pitch, and (**c**) heading angles.

The attitude errors of the four schemes are shown in Figure 11.

It can be seen that the roll error of RAKF was significantly lower compared to RKF at 60 s and the heading error of AKF exhibited larger fluctuations compared to RAKF during the 650–770 s interval. The RAKF scheme showed minimal fluctuations in attitude errors compared to the other three schemes.

To further compare the accuracy of the four filtering approaches, the RMS values of position, velocity, and attitude are summarized in Table 4.

Based on the results shown in Table 4, the following conclusions can be drawn.

Regarding positioning errors, the RAKF scheme achieved positioning accuracy improvements of 45.76%, 30.3%, and 74.27%, respectively, compared to EKF in the north, east, and down directions. The RAKF scheme achieved positioning accuracy improvements of 40.74%, 22.03%, and 59.54%, respectively, compared to AKF. The RAKF scheme achieved positioning accuracy improvements of 3.13% and 5.36% in the north and east direction compared to RKF.

Regarding velocity errors, along the north, east, and down direction, the RAKF exhibits velocity accuracy enhancements of 28.57%, 43.1%, and 65.35%, respectively, compared to EKF. RAKF exhibited velocity accuracy improvements of 21.05%, 34.00%, and 55.7% compared to AKF. RAKF exhibited velocity accuracy improvements of 11.76%, 10.81%, and 12.5% compared to RKF.

Regarding attitude errors, for roll, pitch, and heading angles, RAKF demonstrates attitude accuracy improvements of 55.78%, 45.24%, and 41.41%, respectively, relative to EKF. RAKF demonstrated attitude accuracy improvements of 48.41%, 29.77%, and 37.58% compared to AKF. RAKF demonstrates attitude accuracy improvements of 52.55%, 31.85%, and 32.27% compared to RKF.

By integrating a composite metric of the Position Dilution of Precision (PDOP), number of satellites, quality factor, and position root mean square into the measurement noise covariance matrix, it was found that the enhanced RAKF algorithm effectively suppressed the impact of outlier observations during degraded signal conditions. This method demonstrated exceptional robustness and improved convergence characteristics, significantly boosting the positioning accuracy in GNSS/INS-integrated navigation systems within challenging environments such as urban canyons. Consequently, the system can maintain stable navigation performance under adverse conditions while enhancing the reliability of positioning solutions.

## 4. RBF Neural Network-Aided Robust Adaptive Kalman Filter Testing

In Section 3, the robust adaptive filtering was shown to effectively enhance positioning performance under degraded GNSS conditions. To further validate the adaptability of the RBF neural network-aided Robust Adaptive Kalman Filter (RBF-RAKF) integration scheme during GNSS outages, an additional road test was conducted as detailed below.

### 4.1. Experimental Platform

To reconcile the accuracy requirements for experimental data with practical data acquisition constraints, the GNSS/INS-integrated navigation system experimental platform utilized a Honeywell HGuide N580 high-precision navigation system. The specifications of the device are provided in Table 5.

**Figure 12:**
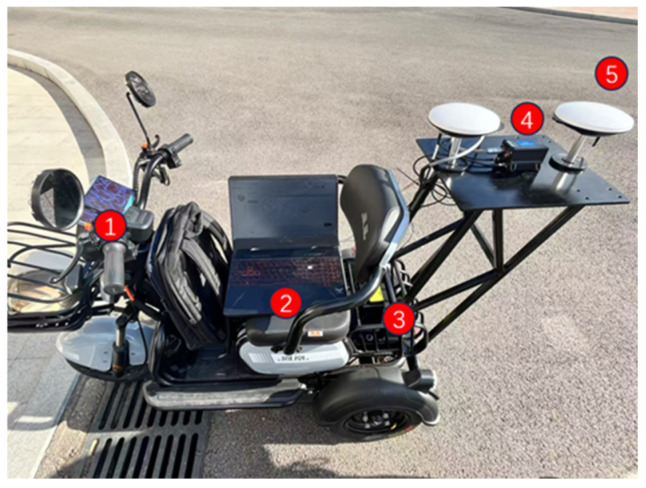
Experimental data collection platform (① a vehicle platform, ② PC control terminal, ③ power supply, ④ IMU, and ⑤ GNSS antenna).

This study employed an electric tricycle as the mobile platform for the GNSS/INS-integrated navigation system. The experimental data collection platform is shown in Figure 12.

### 4.2. Experimental Design

To fully simulate complex scenarios in urban canyon environments and validate the effectiveness of the proposed algorithm, the experimental design incorporated multiple segments of random-duration GNSS signal interruptions to simulate the loss of satellite signals under completely obstructed conditions using a vehicular observation platform. Simultaneously, the test route specifically traversed densely built-up areas with high-rise buildings and sections with severe tree canopy occlusion, reflecting varying obstruction conditions.

The data acquisition was conducted on a designated route within the Shandong University of Science and Technology, encompassing both tree-obstructed and open-sky sections. Five long-distance loop tests were performed on 8 January 2025. The experimental vehicle executed diverse motion patterns including straight-line driving, turning, acceleration, and deceleration to simulate normal vehicle operation.

**Figure 13:**
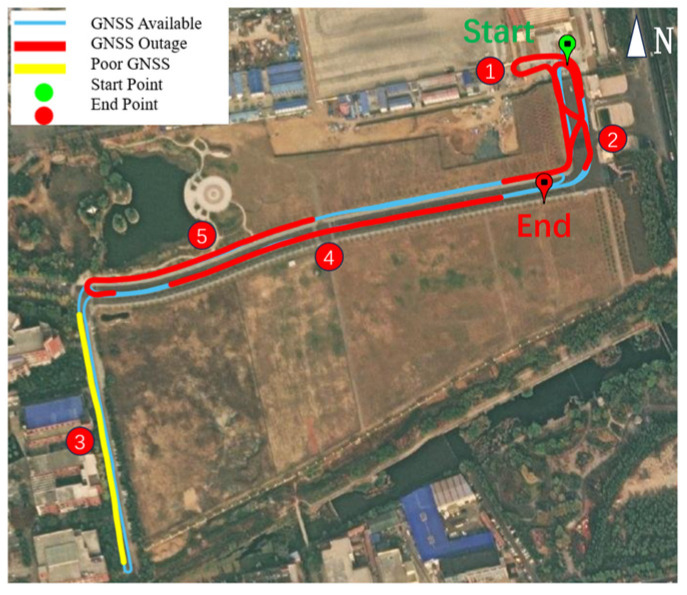
Trajectory and GNSS outage map.

The trajectory and GNSS signal anomalies are illustrated in Figure 13. The entire data collection lasted 1941s, during which, four deliberate GNSS signal interruptions (each lasting 70–90s, highlighted in red on the trajectory map) were introduced to simulate extreme GNSS failure scenarios. The GNSS interruption segments are labeled chronologically as Segments ①, ②, ④, and ⑤. Degraded GNSS signal quality (marked in yellow) was observed from 270,820 to 270,883 s, which was labeled as Segment ③. Notably, a dense tree canopy at location ③ caused substantial satellite signal obstruction.

**Figure 14:**
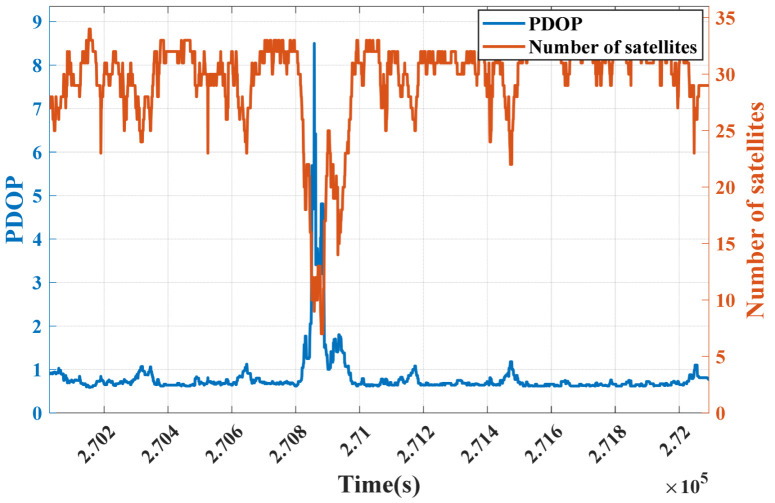
Variation in PDOP and number of satellites.

The variation in PDOP and the number of satellites during the experiment are shown in Figure 14. The data reveal that in open-sky segments, the PDOP values remained low and the number of satellites was over 20 while in severely obstructed areas such as behind Buildings J15 and J13, the PDOP values significantly increased, peaking at 8.5, and the number of satellites dropped to 7–9.

Substantial PDOP fluctuations occurred specifically in GNSS signal interruption and signal-degraded segments, further confirming the impact of environmental obstructions on GNSS positioning performance.

### 4.3. Experimental Results

To systematically validate the effectiveness of the proposed algorithm and conduct an in-depth analysis of performance differences among the various algorithms in the integrated GNSS/INS navigation system, this study designed four distinct algorithmic schemes based on the EKF framework. Each scheme incorporates specific algorithmic enhancement strategies to improve filtering robustness and positioning reliability.

The tightly coupled positioning results post-processed by Inertial Explorer 9.00 software served as the positional reference benchmark. Through comparative analysis of filtering performance across diverse scenarios, this research provides both theoretical foundations and experimental validation for optimizing algorithms in GNSS/INS-integrated navigation systems.

The four algorithmic schemes are as follows:

Scheme 1: Extended Kalman Filter (EKF);

Scheme 2: Robust Adaptive Kalman Filter (RAKF);

Scheme 3: RBF neural network-aided Kalman Filter (RBF-KF);

Scheme 4: RBF neural network-aided Robust Adaptive Kalman Filter (RBF-RAKF).

**Figure 15:**
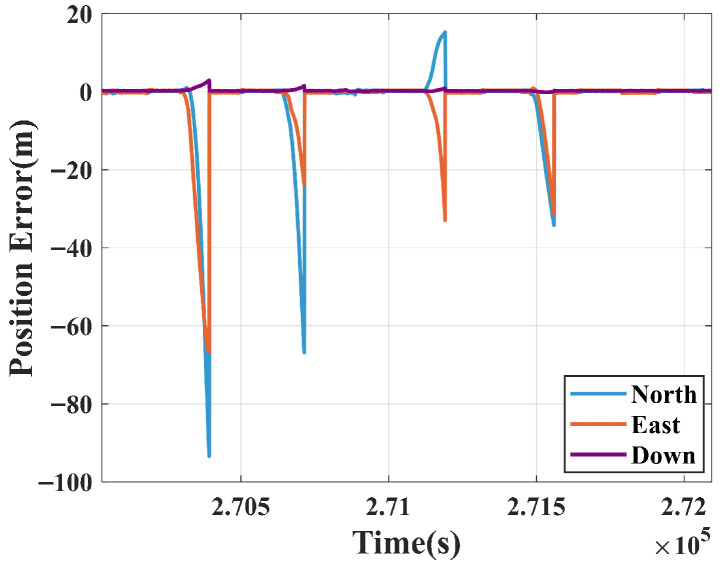
Pure INS solution after GNSS failure.

The positioning error comparison between the traditional Extended Kalman Filter (EKF) and the reference truth under identical GNSS interruption and signal-degraded scenarios is shown in Figure 15.

**Figure 16:**
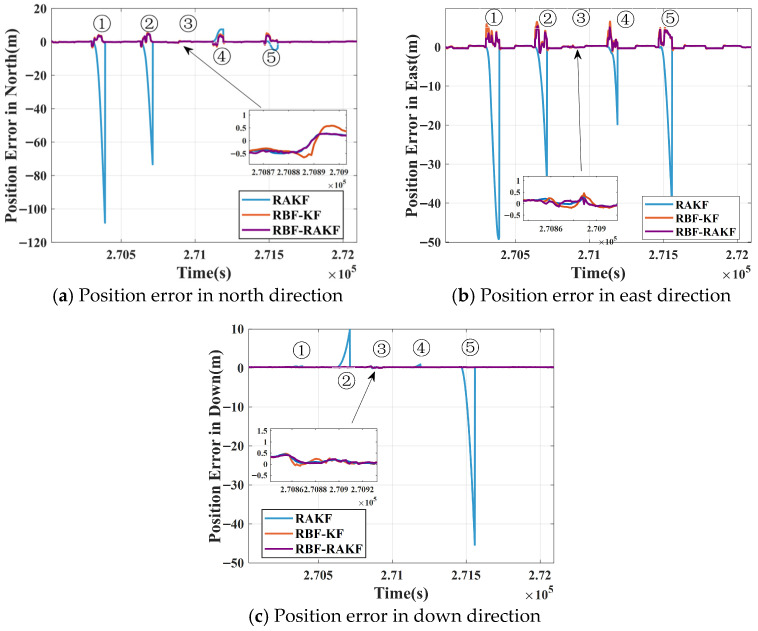
Comparison of positioning errors for three different schemes during GNSS-degraded and interrupted periods in the (**a**) north, (**b**) east, and (**c**) down directions.

The positioning error performance of the three other improved algorithms under identical GNSS interruption and signal-degraded scenarios is shown in Figure 16. Through comparative analysis, the differences in positioning accuracy and stability among these algorithms in complex environments can be visually assessed.

During GNSS signal interruptions (Segments ①, ②, ④, and ⑤), Scheme 1 (EKF) exhibited rapid error accumulation, with particularly pronounced divergence in the north and east directions. For instance, during the first interruption (Segment ①, about 90 s), the errors peaked at 93.4, 66.5, and 2.8 m in the north, east, and down directions, respectively.

In contrast, Scheme 2 (RAKF) dynamically adjusted the measurement noise covariance matrices to suppress error divergence in GNSS-degraded areas. However, it can be seen that it had limited effectiveness during full GNSS interruptions, even demonstrating accelerated divergence in Segments ② and ⑤ (errors reached 110.67, 49.26, and 46.26 m in the north, east, and down directions, respectively).

The algorithms incorporating RBF neural network assistance (Schemes 3–4) demonstrated superior robustness. The peak errors of RBF-KF were 6.32, 6.94, and 0.45 m in the north, east, and down directions, respectively, performance improvements of 89.20%, 85.50%, and 44.65% compared to EKF.

The peak errors of RBF-RAKF were 5.08, 5.67, and 0.44 m in the north, east, and down directions, respectively, performance improvements of91.03%, 88.58%, and 44.71% compared to EKF.

The smallest positioning error fluctuation range was observed with Scheme 4. The smallest positioning errors during GNSS interruptions were observed in GNSS-degraded Segment ③, with Schemes 1 and 3 showing smoother error curves. This outcome confirms that the RBF neural network architecture effectively mitigates INS error accumulation through GNSS pseudo-measurement prediction integrated with kinematic constraint corrections while enhanced robust adaptive filtering suppresses spurious observation impacts.

The error statistics of each algorithm during GNSS-degraded and interrupted periods are summarized in Table 6. From the above figures and tables, it can be observed that the different schemes exhibited varying compensation performances for the integrated navigation system. The RMSEs were 0.94, 1.02, and 0.21 m in the north, east, down directions, respectively. The position accuracy of RBF-RAKF improved by 91.03%, 88.58%, and 44.71% compared to EKF. The position accuracy of RBF-RAKF improved by 92.13%, 87.26%, and 95.41% compared to RAKF. The position accuracy of RBF-RAKF improved by 17.54% and 21.41% in the north and east directions, respectively, compared to RBF-KF.

These results demonstrate that incorporating the improved Robust Adaptive Kalman Filter with the RBF neural network and motion constraints significantly enhances the robustness of GNSS/INS-integrated navigation algorithms in complex urban environments.

The superior performance of Scheme 4 can be attributed to two key mechanisms:

(1) The enhanced RAKF dynamically adjusts the measurement noise covariance matrix based on parameters like PDOP and the number of satellites. This reduces the weight of abnormal observations during GNSS signal fluctuations, effectively preventing filter divergence.
(2) During the period when GNSS signals are available, the RBF neural network trains using high-precision navigation outputs from the improved Robust Adaptive Kalman Filter. During GNSS outages, INS data is used to predicts pseudo-position increments through the trained model of the RBF neural network. This scheme significantly suppresses the exponential accumulation of INS errors.

## 5. Conclusions

In this study, an RBF neural network-aided robust adaptive GNSS/INS-integrated navigation algorithm was proposed. It employs a multi-factor dynamically adjusted measurement noise covariance matrix within the RAKF to enhance positioning accuracy under poor GNSS signal conditions while simultaneously establishing an RBF neural network training model. During GNSS outages, the model predicts GNSS pseudo-measurements to ensure navigation and positioning precision. It can be seen that the RBF neural network-aided Robust Adaptive Filter (RBF-RAKF) achieved the highest error compared to the other schemes. The RMSEs were 0.94, 1.02, and 0.21 m in the north, east, and down directions, respectively. The position accuracy of RBF-RAKF improved by 91.03%, 88.58%, and 44.71% compared to the traditional Extended Kalman Filter (EKF). Crucially, the algorithm maintained meter-level stability under vegetation and building obstructions, with a horizontal positioning RMS error better than 1.5 m and a vertical error RMS below 0.3 m.

In the future, the following areas can be improved. The training effectiveness of RBF neural networks depends on the quality of GNSS data. During prolonged GNSS signal outages, the prediction accuracy of neural networks may decrease due to insufficient training data.

In summary, a combination of theoretical analysis and experimental validation verified the engineering applicability of the proposed algorithm in complex urban environments. The proposed algorithm effectively suppresses the impact of abnormal observations on the system when observation quality deteriorates, demonstrating superior robustness and excellent filter convergence characteristics. It also effectively curbs INS error accumulation during GNSS signal interruptions. This work provides a reliable technical foundation for the practical application of high-precision navigation systems in dynamic signal-obscured scenarios.

## Figures and Tables

**Table 1:** Specifications of sensors.

Performance Parameter	Value
Gyroscope Bias	0.25°/h
Angular Random Walk	0.04°/√h
Accelerometer Bias	0.025 mg
Velocity Random Walk	0.03 m/s/√h

**Table 2:** Values of measurement noise covariance matrix.

Scheme	*R* Value
Scheme 1	$R=r^{2}$
Scheme 2	$R=(1+PDOP\times \gamma )*r^{2}$
Scheme 3	$R=(1+Q)\times r^{2}$
Scheme 4	$R=(1+Nsat\times \eta )\times r^{2}$
Scheme 5	$R=(1+PDOP\times \gamma +Q)\times r^{2}$
Scheme 6	$R=(1+PDOP\times \gamma +Nsat\times \eta )\times r^{2}$
Scheme 7	$R=(1+Nsat\times \eta +Q)\times r^{2}$
Scheme 8	$R=(1+PDOP\times \gamma +Nsat\times \eta +Q)\times r^{2}$

**Table 3:** RMS of position, velocity, and attitude with different measurement noise covariance matrices.

	Position (m)			Velocity (m/s)			Attitude (deg)		
	$P_{N}$	$P_{E}$	$P_{D}$	$V_{N}$	$V_{E}$	$V_{D}$	Roll	Pitch	Heading
Scheme 1	0.0590	0.0660	0.2060	0.0420	0.0580	0.1010	0.1470	0.1680	0.3260
Scheme 2	0.0420	0.0481	0.1175	0.0311	0.0415	0.0669	0.1214	0.1426	0.2468
Scheme 3	0.0422	0.0484	0.1260	0.0311	0.0416	0.0712	0.1220	0.1439	0.2469
Scheme 4	0.0420	0.0481	0.1167	0.0310	0.0414	0.0662	0.1215	0.1425	0.2461
Scheme 5	0.0425	0.0485	0.1280	0.0313	0.0417	0.0715	0.1221	0.1438	0.2469
Scheme 6	0.0422	0.0483	0.1189	0.0312	0.0415	0.0671	0.1215	0.1424	0.2461
Scheme 7	0.0425	0.0485	0.1273	0.0312	0.0417	0.0714	0.1221	0.1438	0.2463
Scheme 8	0.0417	0.0480	0.1152	0.0309	0.0414	0.0666	0.1213	0.1427	0.2468
Improved (%)	29.32%	27.27%	44.07%	26.43%	28.62%	34.06%	17.48%	15.06%	24.3%

**Table 4:** RMS of position, velocity, and attitude of different algorithms.

	Position Errors (m)			Velocity Errors (m/s)			Attitude Errors (deg)		
	N	E	D	N	E	D	Roll	Pitch	Heading
EKF	0.059	0.066	0.206	0.042	0.058	0.101	0.147	0.168	0.326
AKF	0.054	0.059	0.131	0.038	0.050	0.079	0.126	0.131	0.306
RKF	0.033	0.046	0.056	0.034	0.037	0.040	0.137	0.135	0.282
RAKF	0.032	0.046	0.053	0.030	0.033	0.035	0.065	0.092	0.191

**Table 5:** Specifications of Honeywell HGuide N580 high-precision navigation system.

Performance Parameter	Value
Accelerometer Bias	0.025 mg
Gyroscope Bias	0.25°/h
Velocity Random Walk	0.03 m/s/√h
Angular Random Walk	0.04°/√h
IMU Sampling Rate	100 Hz
GNSS Sampling Rate	10 Hz

**Table 6:** RMS of positioning using different schemes.

Scheme	North (m)	East (m)	Down (m)
EKF	10.52	8.96	0.39
RAKF	11.90	8.01	4.58
RBF-KF	1.14	1.30	0.22
RBF-RAKF	0.94	1.02	0.21

## Data Availability

The data presented in this study are available on request from the corresponding author.
